# Azilsartan: Current Evidence and Perspectives in Management of Hypertension

**DOI:** 10.1155/2019/1824621

**Published:** 2019-11-03

**Authors:** Akshyaya Pradhan, Ashish Tiwari, Rishi Sethi

**Affiliations:** Department of Cardiology, King George's Medical University, Lucknow, India

## Abstract

Hypertension continues to be global pandemic with huge mortality, morbidity, and financial burden on the health system. Unfortunately, most patients with hypertension would eventually require two or more drugs in combination to achieve their target blood pressure (BP). To this end, emergence of more potent antihypertensive drugs is a welcome sign. Angiotensin receptor blockers (ARBs) are cornerstones of hypertension management in daily practice. Among all ARBs, azilsartan is proven to be more potent in most of the head-to-head trials till date. Azilsartan is the latest ARB approved for hypertension with greater potency and minimal side effects. This review highlights the role of azilsartan in management of hypertension in the current era.

## 1. Introduction

Hypertension continues to be a global health pandemic causing huge mortality and morbidity. It contributes for almost half of CVD and stroke deaths [1, 2]. Unfortunately, it is no more a disease of western world and in South Asia itself, almost one third of population is suffering from hypertension [3]. To compound the situation, the recent NHANES data peg the disease treatment rates at only 71–80%, whereas the overall control rates are dismal at 45–50% [4]. More importantly, a majority of them would require two or more drug combinations to achieve their blood pressure goals [5, 6].

**Figure 1:**
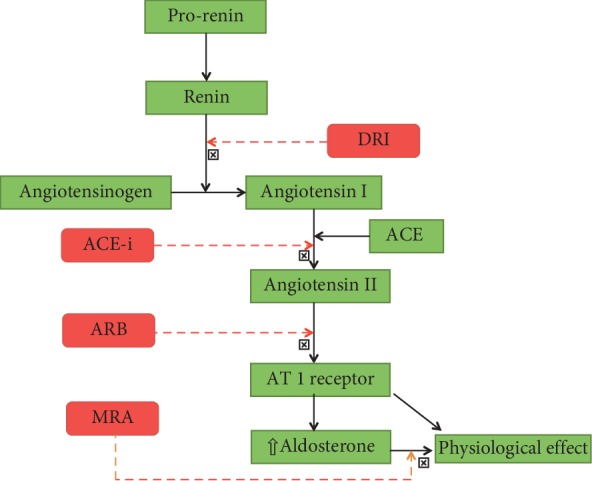
Drugs acting on renin angiotensin aldosterone system. ACE, angiotensin-converting enzyme; ACE-i, angiotensin-converting enzyme inhibitor; ARB, angiotensin receptor blocker; DRI, direct renin inhibitor; MRA, mineralocorticoid receptor antagonist; and AT1, angiotensin 1.

Drugs targeting the renin-angiotensin-aldosterone system (RAAS) are cornerstone of the management of hypertension. Four classes of molecules make it to the list of RAAS blockers: angiotensin-converting enzyme (ACE) inhibitors, angiotensin receptor blockers (ARBs), mineralocorticoid receptor antagonist, and direct renin inhibitors (DRI) (Figure 1). Aldosterone antagonists are primarily reserved for resistant hypertension, whereas major trials of DRI did not meet their primary end points. Hence, RAAS modulators in daily practice of hypertension include ACEi and ARB. Because of a favourable side effect profile, many practitioners choose ARB over ACEi as first-line therapy.

## 2. Evolution of ARBs in Hypertension

**Figure 2:**
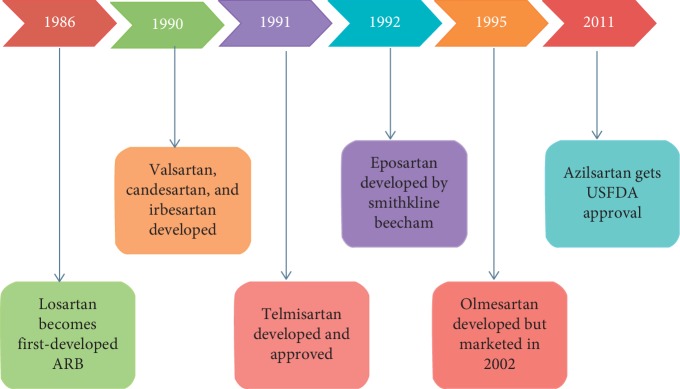
Milestones of development of various ARB's. Eprosartan was another ARB developed in 1992 by Glaxo Smithkline but not marketed in our country though approved by USFDA; losartan got USFDA approval only in 1995.

ARBs act via inhibiting the angiotensin II type 1 receptor and decreasing RAAS-associated adverse effects. The first ARB which was approved for hypertension was losartan, way back in 1986 [7]. Till March 2018, Food and Drug Administration (FDA) approved 8 ARBs for various indications. In chronological order the list includes losartan, valsartan,candesarten, irbesartan,eposartan, telminsartan, olmesartan, and azilsartan, being the latest addition (Figure 2).

As ARBs cause dose-dependent decrease in peripheral resistance, it decreases the effect of aldosterone on the kidney and peripheral vasculature including decrease in smooth muscle vascular tone. ARBs have been successfully used in management of hypertension, coronary heart disease, heart failure, chronic kidney disease, and other miscellaneous conditions. Because of difference in affinity for the angiotensin receptor and other mechanisms, various ARBs differ in their pharmacokinetics and pharmacodynamics in human body. Major trials of different ARBs approved in our country for treating hypertension are LIFE (Losartan), ROADMAP (Olmesartan), VALUE (Valsartan), and ON TARGET (Telmisartan) [8–11] (see Table 1).

## 3. Azilsartan: ARB with a Difference

Azilsartan medoxomil (development code: TAK-491) has been developed by Takeda Global Research & Development Centre, Inc., U.S. and got FDA approval in February 2011 for treatment in hypertension in adults [12]. Azilsartan is now worldwide approved for hypertension either as a prodrug (Azilsartan medoxomil) or primary compound.

## 4. Mechanism of Action, Pharmacodynamics, and Pharmacokinetics

Azilsartan medoxomil is a prodrug which is hydrolysed in the gastrointestinal tract before getting absorbed in the system. Azilsartan acts against angiotensin II in a dose-dependent manner. After administration of azilsartan to healthy subjects, plasma angiotensin I and II concentrations increased, while plasma renin activity increased while plasma aldosterone concentrations decreased. Azilsartan does not cause any clinical significant effects on serum sodium or potassium. After oral administration, bioavailability of azilsartan medoxomil is approximately 60% with peak plasma concentration reached within 1.5 to 3 hours. There is no food interaction on bioavailability of azilsartan [13].

Azilsartan is closely related to candesartan with greater potency and prolonged duration of action as compared with other ARBs. Unlike candesartan which must be orally administrated as the prodrug (candesartan cilexetil) for better bioavailability, azilsartan is equally effective as either ester prodrug (azilsartan medoxomil) or primary compound itself. Azilsartan contains an oxo-oxadiazole ring which is not found in any of clinically approved ARBs, which makes azilsartan less acidic and more lipophilic than others.

## 5. Comparison with Other Sartans: Clinical Evidence

**Figure 3:**
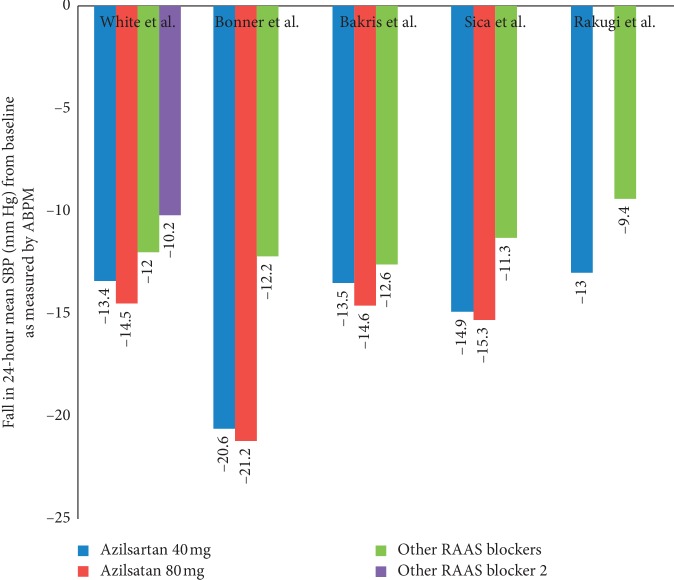
Head-to-head comparison of azilsartan and other RAAS blockers in clinical studies for reduction in 24-hour mean systolic blood pressure (SBP, as measured by ambulatory BP monitoring) from baseline. RAAS blockers used as a comparator arm in various studies were as follows: White et al., valsartan 320 mg (purple bar) and olmesartan 40 mg (green bar); Bonner et al., ramipril 10 mg; Bakris et al., olmesartan 40 mg; Sica et al., valsartan 320 mg; Rakugi et al., candesartan.

All major head-to-head randomized controlled trials indicate that azilsartan exhibits more potent antihypertensive action than any other drugs in its class. This potent antihypertensive action includes better clinical systolic blood pressure (SBP), diastolic blood pressure (DBP), and 24 hour ambulatory blood pressure (Figure 3, Table 2).

In a randomized, double-blinded, placebo-controlled trial, Sica et al. compared azilsartan medoxomil and valsartan in primary hypertension using ambulatory and clinic BP measurement [13]. In the trial, azilsartan 40 mg (−14.9 mm Hg) and 80 mg (−15.3 mm Hg) significantly improved 24-hour mean SBP than valsartan (−11.3 mm Hg; *p* < 0.001).

Bakris et al. compared azilsartan medoxomil with olmesartan medoxomil in 1275 primary hypertension patients. Azilsartan 80 mg (−14.6 mm Hg) significantly improved mean SBP versus olmesartan (−12.6 mm Hg; *p*=0.038), whereas azilsartan 40 mg (−13.5 mm Hg) dose was noninferior to olmesartan. In this study, azilsartan was well tolerated and more efficacious at its maximal dose than the highest dose of olmesartan medoxomil [14].

White et al. compared azilsartan 40–80 mg with valsartan 320 mg and olmesartan 40 mg in a double-blinded, placebo-controlled RCT [15]. Trial results revealed that azilsartan 80 mg (−14.5) significantly improved mean SBP more than olmesartan (−11.7) and valsartan (−10.2). Azilsartan medoxomil at 40 mg (−13.4 mm Hg) was also noninferior to 40 mg of olmesartan (−1.4 mm Hg).

Another RCT comparing azilsartan 20–40 mg versus candesartan 8–12 mg by Rakugi et al. showed significantly improved DBP (−12.4 vs. −9.8; *p*=0.0003) and SBP (−21.8 vs. −17.5; *p* < 0.0001) with azilsartan as well as 24 hour ambulatory blood pressure [16].

The prospective, observational, multicentre EARLY registry in Germany compared patients initiated on monotherapy comprising either azilsartan or an ACE-inhibitor [17]. The results revealed that azilsartan medoxomil provided statistically significant albeit small improvement in blood pressure control. More patients on azilsartan attained blood pressure targets vis-a-vis ACE inhibitors (61.1% vs. 56.4%; *p* < 0.05).

Finally, Takagi et al. performed a meta-analysis which included a total of 6152 patients from 7 randomized-controlled trials with azilsartan [18]. The pooled analysis suggested a significant reduction in BP changes among patients randomized to 40 mg of azilsartan versus control therapy (clinic SBP: −4.20 mm Hg; 95% CI: −6.05 to −2.35 mm Hg; *p* < 0.00001; clinic DBP: −2.58 mm Hg; 95% CI: −3.69 to −1.48 mm Hg; *p* < 0.00001; 24-h mean SBP: −3.33 mm Hg; 95% CI: −4.74 to −1.93 mm Hg; *p* < 0.00001; and 24-h mean DBP: −2.12 mm Hg; 95% CI: −2.74 to −1.49 mm Hg; *p* < 0.00001). Meta-analysis concluded that in patients with hypertension, azilsartan therapy resulted in greater BP reduction.

## 6. Azilsartan in Combination Therapy

**Figure 4:**
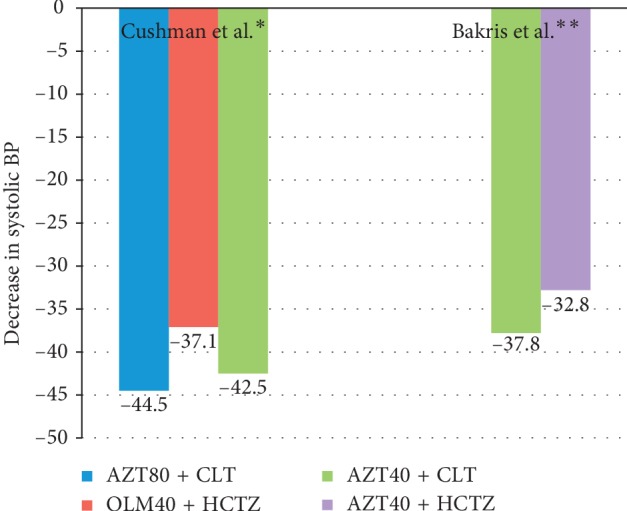
Clinical studies of head-to-head comparison of combination therapy with azilsartan for reduction in mean systolic blood pressure from the baseline. ^*∗*^24-hour systolic BP measured by ABPM; ^*∗∗*^clinic systolic BP used; AZT, azilsartan; CLT, chlorthalidone; HCTZ, hydrochlorothiazide; and OLM, olmesartan.

There are a few studies available in the literature comparing azilsartan-based combination therapies and most of them have used azilsartan in combination with chlorthalidone. In the largest such study, Cushman et al. compared azilsartan (40/80 mg) and chlorthalidone combination with olmesartan (40 mg) and hydrochlorothiazide combination [20]. They enrolled 1071 patients with stage 2 hypertension and evaluated mean ABPM (Systolic BP) pressure at 12 weeks. Azilsartan-based combination therapies lowered systolic BP (ABPM) better than olmesartan-based regimens (*p* < 0.001 for all comparisons, Figure 4).

Similarly, Bakris et al. compared an azilsartan plus chlorthalidone regimen with azilsartan with hydrochlorothiazide in 609 patients with moderate-to-severe hypertension [21]. The dose of azilsartan was 40 mg while diuretic doses were titrated from 12.5 mg to 25 mg. The fall in clinical systolic BP from the baseline was higher in the chlorthalidone-based regimen (–37.8 mm Hg vs. –32.8 mm Hg, respectively, *p* < 0.001).

Interestingly, the blood pressure-lowering efficacy was maintained across both white and black races as demonstrated by Ferdinand et al. [22]. In this pooled analysis from two RCTs, azilsartan-based combination as well as monotherapy resulted in better BP control among both black and whites alike when pitted against an olmesartan-based regimes.

## 7. Azilsartan: Effects beyond Blood Pressure Control

Because of its inverse agonistic effects, azilsartan has potential effects beyond BP control which include amelioration of deleterious effects of angiotensin II such as cardiac hypertrophy, fibrosis, insulin resistance, and stabilization of coronary plaques [23].

In patients with heart failure with preserved ejection fraction (HfpEF), azilsartan improved parameters of diastolic function of left ventricle [24]. In their study of fifteen patients with HfpEF by Sakomoto et al., mitral annular E/e' ratio on echocardiography decreased with azilsartan therapy at six months while there was no change with candesartan treatment. This was despite comparable with reductions in blood pressure with both drugs. Azilsartan also decreased heart rate in the study while candesartan did not.

Azilsartan also improved endothelial dysfunction better than amlodipine as assessed by flow-mediated dilatation in brachial artery. In a group of twenty four hypertensive patients, 3 months of azilsartan therapy achieved superior flow-mediated dilatation, higher plasma renin activity, and lower plasma aldosterone levels [25]. Azilsartan therapy was also associated with improvement in arterial stiffness parameters (assessed by carotid-femoral pulse wave velocity) at 6 months [26].

In the CHAOS study published by Sezai et al., the effect of azilsartan and olmesartan on plasma renin activity, aldosterone II, and angiotensin in patients with essential hypertension after cardiac surgery was studied [27]. Apart from the primary endpoint, CHAOS study also included left ventricular mass index (LVMI), estimated glomerular filtration rate (eGFR), and urinalysis as secondary end point. The plasma renin levels were not different between the groups but aldosterone and angiotensin II levels were lower with olmesartan arm. There was no difference in two groups in terms of eGFR and urinalysis. LVMI was significantly lower in the olmesartan group than in the azilsartan group (*p* < 0.0001).

A cost effectiveness analysis between various ARB-based combination therapy has been published [28]. Azilsartan plus chlorthalidone combination therapy proved to have maximal incremental cost effectiveness followed by losartan plus hydrochlorthiazide-based therapy. Because of increased efficacy, azilsartan-based group was dominant despite an increment in price.

## 8. Side Effect of Drug

The various side effects of the drug seen in clinical studies include dizziness (8.9%), increased serum creatinine (3.6%), fatigue (2%), diarrhoea (2%), hypotension (1.7%), and syncope (0.3%) [12, 29]. Hypotension was the commonest cause of drug discontinuation with monotherapy, whereas raised serum creatinine and dizziness were most abundant causes in combination with chlorthalidone. Other side effects reported by the manufacturer include fatigue, muscle spasm, nausea, and abnormalities in hemogram. Increases in serum creatinine were often transient and related to a large fall in blood pressure. They were exacerbated by old age (>75 years) as well as coadministration of diuretics. On a similar note, manufacturers warn that volume and salt-depleted individuals are more prone to hypotensive effects of the drug. As with other ARBs, it should not be administered during pregnancy.

## 9. Current Indications

ARB has the best patient satisfaction profile (as assessed by the lowest rate of treatment discontinuation) among contemporary drugs [30]. With plethora of data establishing the superiority of azilsartan in controlling BP, it can be recommended that wherever blood pressure is not controlled on combination therapy with or without ARBs, adding azilsartan or replacing other ARBs with azilsartan can be an acceptable approach. For *de novo* hypertension too, azilsartan is an attractive option. However, for patients whose blood pressure is already well controlled by other ARBs, it is not imperative to switch to azilsartan. The drug is listed as the first-line ARB in the recent ACC/AHA 2017 Hypertension guidelines [5]. Azilsartan was also used in the pivotal SPRINT trial which has redefined blood pressure goals [31]. Based on the data from the available clinical trials, the dose equivalence between azilsartan and other ARBs is summarized in Table 3.

## 10. Conclusion

**Figure 5:**
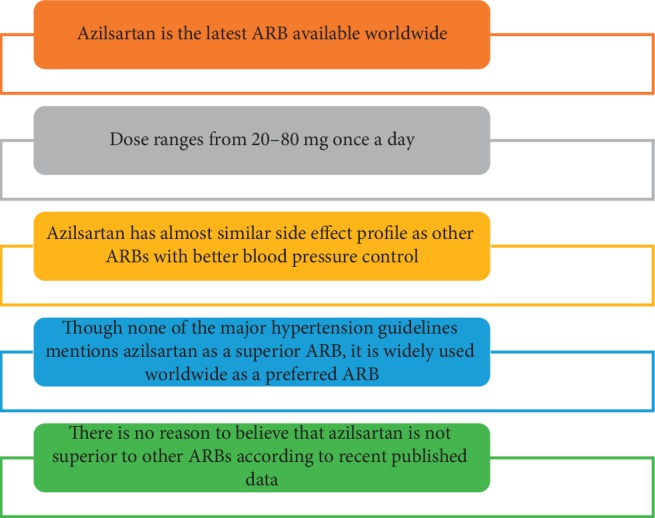
Take home messages.

Hypertension is a global pandemic with huge morbidity and mortality. Unfortunately, the awareness and control rates remain dismal even in the western world. Azilsartan is the latest ARB to be added to the armamentarium of hypertension (Figure 5). It has emerged as a potent ARB which has demonstrated superior BP control in both monotherapy and combination therapy not only against other ARBs but also against other class of antihypertensive agents. The drug becomes the first-line choice in patients whose BP is not at goal despite combination therapy. In *de novo* hypertension, ARB is often the first-line choice due to their better tolerability and potency. In this scenario, it is the discretion of the treating physician to initiate any ARB of his choice but the wealth of data discussed above makes azilsartan an attractive first-line ARB.

## Figures and Tables

**Table 1:** Pivotal trials of ARB's and their key findings.

ARB	Major trials	Number of patients	Year	Major findings
Losartan	LIFE [8]	9193	2002	Losartan prevents more cardiovascular morbidity and death than atenolol for similar reduction in blood pressure and is better tolerated. Losartan seems to confer benefits beyond reduction in blood pressure

Telmesartan	ONTARGET [9]	25,620	2008	Telmisartan was equivalent to ramipril in patients with vascular disease or high-risk diabetes and was associated with less angioedema. The combination of the two drugs was associated with more adverse events without an increase in benefit

Olmesartan	ROADMAP [10]	1147	2015	Additive treatment with an angiotensin receptor blocker, olmesartan, did not improve clinical outcome in hypertensive patients with chronic heart failure (CHF) treated with angiotensin-converting enzyme (ACE) inhibitors, *β*-blockers, or both

Valsartan	VALUE [11]	15,425	2004	The hypothesis with equivalent amount of blood pressure control, valsartan would reduce cardiac morbidity and mortality more than amlodipine in hypertensive patients with high cardiovascular risk could not be proved Unequal reductions in blood pressure might account for differences between the groups in cause-specific outcomes. The findings emphasise the importance of prompt blood-pressure control in hypertensive patients with high cardiovascular risk

**Table 2:** Major azilsartan studies and their results.

	Major trials/studies of azilsartan						
	Design	Number of patients	Inclusion criteria	Duration	Dose	Primary outcome	Results
Sica et al. [13]	RCT, double blinded, placebo controlled	984	SBP 150–180 mm Hg and 24-hour mean SBP 130–170 mm Hg	24 weeks	Azilsartan 40 or 80 mg OD vs. valsartan 320 mg OD	Change in 24-hour mean SBP by ABPM from baseline	Azilsartan 40 mg (−14.9) and 80 mg (−15.3) significantly improved 24-hour mean SBP (−11.3) *p* < 0.0001

Bakris et al. [14]	RCT, double blinded, placebo controlled	1275	SBP 150–180 mm Hg or 24-hour mean SBP 130–170 mm Hg	6 weeks	Azilsartan 20, 40, 80 mm Hg OD vs. olmesartan 40 mg OD vs. placebo	Change in 24-hour mean SBP by ABPM from baseline	Azilsartan 80 mg (−14.6) significantly improved mean SBP vs. olmesartan (−12.6) (*p*=0.038) 40 mg dose was noninferior to olmesartan
White et al. [15]	RCT, double blinded, placebo controlled	1291	SBP 150–180 mm Hg and 24-hour mean SBP 130–170 mm Hg	6 weeks	Azilsartan 40, 80 mg OD vs. olmesartan 40 mg OD vs. valsartan 320 mg OD	Change in 24-hour SBP by ABPM from baseline	Azilsartan 80 mg (−14.5) significantly improved mean SBP more than olmesartan (−11.7) and valsartan (−10.2). Azilsartan 40 mg (−13.4) noninferior to olmesartan

Rakugi et al. [16]	RCT, double blinded, placebo controlled	622	Grade I-II essential hypertension	16 weeks	Azilsartan 20–40 mg OD vs. candesartan 8–12 mg OD	Change in sitting SBP, DBP, and ABPM	Azilsartan significantly improved DBP (−12.4) vs. candesartan (−9.8) (*p*=0.0003) and SBP azilsartan (-21.8) vs. candesartan (−17.5) (*p* < 0.0001)

Gitt et al. (EARLY registry) [17]	Prospective, observational, national, multicenter registry	3849	>18 year, essential hypertension	12 months	Azilsartan 40 and 80 mg vs. ACE-inhibitor (mainly ramipril 10 mg)	Change in clinic SBP, DBP, and ABPM	Azilsartan 40 and 80 mg reduced both clinic systolic BP and mean ambulatory systolic BP significantly more than ramipril at a dose of 10 mg. Clinic SBP −20.6 + −0.9 with 40 mg and −21.2 ± 0.9 with 80 mg Vs. ramipril −12.2 ± 0.9

Takagi et al. [18]	Meta-analysis	6152	Essential hypertension	—	Azilsartan 40 mg vs. control	Change in SBP and DBP	SBP reduction difference −4.2 mm Hg; DBP reduction difference −2.58; SBP (ABPM) −3.33 mm Hg; DBP (ABPM) −2.12 mm Hg (*p* < 0.0001)

Kario et al. [19]	RCT	668	Stage I and II hypertension^*∗*^	8 weeks	Azilsartan 20 mg vs. amlodipine 5 mg	Sleep ABPM	Among those >60 years, similar control rate of sleep BP, despite a trend favouring amlodipine (35% vs. 30%)

**Table 3:** Dose equivalence of azilsartan with other sartans based on available data.

Dose equivalence of azilsartan to other ARBs		
Azilsartan	Valsartan 320 mg	Sica et al. [13]
Azilsartan	Olmesartan 40 mg	White et al. [15]
Azilsartan	Ramipril 10 mg	Gitt et al. [17]
Azilsartan	Amlodipine 5 mg	Kario and Hoshide [19]
